# Artificial Intelligence in Histopathological Analysis for Predicting Immunotherapy Response in Cutaneous Melanoma

**DOI:** 10.3390/ijms262110729

**Published:** 2025-11-04

**Authors:** Seungah Yoo, Ji Hyun Lee

**Affiliations:** Department of Dermatology, Seoul St. Mary’s Hospital, College of Medicine, The Catholic University of Korea, Seoul 06591, Republic of Korea

**Keywords:** artificial intelligence, melanoma, pathology, tumor-infiltrating lymphocytes, tertiary lymphoid structures, immunotherapy

## Abstract

Artificial intelligence (AI) has emerged as a transformative tool in histopathology, offering new opportunities to enhance prognostic accuracy and guide immunotherapy in cutaneous melanoma. The prognostic significance of tumor-infiltrating lymphocytes (TILs) is well established, yet their manual assessment remains subjective, labor-intensive, and often confined to selected tissue regions. Recent AI-based approaches enabled automated and reproducible quantification of TIL density and spatial immune profiling across whole-slide images, providing a more comprehensive view of the tumor immune microenvironment. In melanoma, these methods have demonstrated the potential to predict response to immune checkpoint blockade, with spatially resolved TIL profiling emerging as a particularly powerful prognostic and predictive biomarker. This review summarizes recent advances in AI-driven histopathologic analysis of cutaneous melanoma, focusing on automated TIL quantification and spatial immune profiling, and highlights how these innovations refine prognostic evaluation and improve the prediction of immunotherapy outcomes.

## 1. Introduction

### 1.1. Cutaneous Melanoma and the Emergence of Immunotherapy

Cutaneous melanoma is a highly aggressive form of skin cancer originating from melanocytes. Although it accounts for only about 1% of all skin cancers, it is responsible for nearly 80% of skin cancer-related deaths [1,2]. Patients with advanced or unresectable disease still face poor outcomes, with a 5-year survival of 22.5% in stage IV disease [3]. The advent of immune checkpoint inhibitors (ICIs), particularly those targeting programmed cell death protein 1 (PD-1), has significantly reshaped the therapeutic landscape and improved survival in many cases. However, a considerable fraction of patients fail to benefit, and combination regimens such as dual checkpoint inhibition often bring substantial toxicity alongside modest gains [4,5,6]. This unpredictable balance between benefit and risk underscores the pressing need for reliable biomarkers to guide treatment decisions.

### 1.2. Limitations of Current Biomarkers for Immunotherapy in Melanoma

Current biomarkers have not fully met this need. Tumor mutational burden (TMB), a predictive biomarker in various cancers, has shown inconsistent and relatively limited reliability in melanoma [7]. TMB alone is often insufficient to differentiate responders from non-responders in melanoma, as treatment outcomes are influenced by additional factors such as neoantigen clonality, copy number alterations, and mutations in antigen presentation pathways [8]. Its predictive value in melanoma is often reported only when using thresholds far higher than the FDA’s approved cutoff of 10 mut/Mb, sometimes exceeding 50 or even 100 mut/Mb [9,10,11,12]. Its usefulness may also depend on patient factors such as sex, or on whether ICIs are given as monotherapy versus in combination [13,14].

Similarly, programmed death-ligand 1 (PD-L1) expression exhibits variability across patients, between primary and metastatic sites, and even within the same tumor [15,16,17]. The lack of standardized assays and diverse cutoffs, ranging from 1% to 50%, further complicates interpretation [18]. Notably, many PD-L1-negative patients still respond to immunotherapy [19]. These limitations reduce the reliability of TMB or PD-L1 as independent predictive biomarkers in melanoma.

### 1.3. The Potential of AI in Enhancing Biomarker Discovery

Recently, artificial intelligence (AI) is transforming many fields of medicine, including oncology. Ciresan et al. demonstrated that deep neural networks could accurately detect mitoses in breast cancer histology, introducing the idea that neural networks might serve as a viable approach for interpreting cancer pathology images. Esteva et al. later extended this concept to clinical dermatology by training a convolutional neural network (CNN) on skin lesion images, achieving dermatologist-level performance in classifying melanoma and other skin cancers [20,21]. These pioneering studies established the groundwork for applying AI to medical image analysis.

Among AI technologies, deep learning, particularly CNNs, has achieved performance comparable to that of human experts in image interpretation [22,23,24]. In pathology, the digitization of slides into whole-slide images (WSIs) enable AI to analyze entire tissue sections rather than selected areas. This facilitates automated detection of tumor regions, systematic quantification of immune infiltrates such as tumor-infiltrating lymphocytes (TILs), and consistent characterization of the tumor microenvironment (TME) [25]. Beyond efficiency, these methods hold promise for identifying biomarkers that capture the biological complexity underlying diverse cancers and treatment responses.

### 1.4. Scope of This Review

This review summarizes recent advances in AI-driven pathology for melanoma, with particular emphasis on automated assessment of TILs. As a narrative review, it may be subject to selection bias. We highlight how these emerging techniques can refine prognostic and predictive stratification, discuss methodological progress, and outline the challenges to clinical implementation.

## 2. Tumor Microenvironment (TME) and TILs

### 2.1. The Central Role of TME and Emergence of TILs

The recognition of the TME as a key determinant of cancer initiation, progression, and therapy response has shifted our view of cancer from a cell-centered disease to a dynamic ecosystem [26,27]. Within this complex ecosystem, diverse stromal and immune cells, including tumor-associated macrophages (TAMs), myeloid-derived suppressor cells (MDSCs), and cancer-associated fibroblasts (CAFs), influence the evolution and response of tumors to treatment. ICIs act in part by reprogramming this environment, underscoring the importance of TILs as both effectors of antitumor immunity and potential biomarkers [28,29,30].

### 2.2. Prognostic and Predictive Value in Melanoma

Across cancers, abundant TILs are generally associated with favorable clinical outcomes and stronger responses to ICIs [31,32]. Compared to TMB and PD-L1, TIL density (CD8+ cell count/mm^2^) has shown superior performance in predicting responses to neoadjuvant immunotherapy [33]. These findings reinforce the potential of TILs as a more consistent biomarker.

In melanoma, higher levels of CD3+ and CD8+ T cells, CD4+ helper T cells, and CD20+ B cells are consistently correlated with a favorable prognosis, whereas increased FOXP3+ regulatory T cells typically indicate poorer outcomes [34]. Likewise, the presence of activated T cells, B cells, and mature dendritic cells at the invasive margin is associated with prolonged survival, while infiltration by plasmacytoid dendritic cells or neutrophils tends to signify more aggressive disease [35]. Compared with conventional markers such as TMB or PD-L1, the density and composition of TILs often provide more reliable indicators of therapeutic response, positioning them as strong candidates for clinical biomarkers.

### 2.3. Barriers to Clinical Implementation

From a diagnostic perspective, profiling the distribution of immune cells, including TILs, can help stratify tumors according to their immune status. From a therapeutic standpoint, such stratification enables more accurate evaluation of immunotherapy response and potential toxicity, facilitating more personalized treatment strategies. However, several challenges limit routine clinical application of TIL analysis:

- Whole-slide analysis: Capturing the full complexity of the TME requires analysis of entire WSIs. Manual review of WSIs is prohibitively time-consuming and labor-intensive, often leading to reliance on selected regions of interest (ROIs) and a risk of missing spatial heterogeneity [36,37].
- Spatial context: Beyond simple cell counts, the spatial arrangement of TILs relative to other cells carries prognostic significance. Integrating these spatial patterns improves predictive accuracy but adds technical complexity [38,39].
- Standardization of cutoffs: Although numerous studies have confirmed the favorable impact of higher TIL densities, translating this finding into clinical practice requires harmonized criteria. Defining clinically meaningful, tumor-specific cutoff values is essential for consistent patient stratification [40].

## 3. AI-Based Quantification and Analysis of TILs

### 3.1. From Manual to Automated Assessment

**Figure 1:**
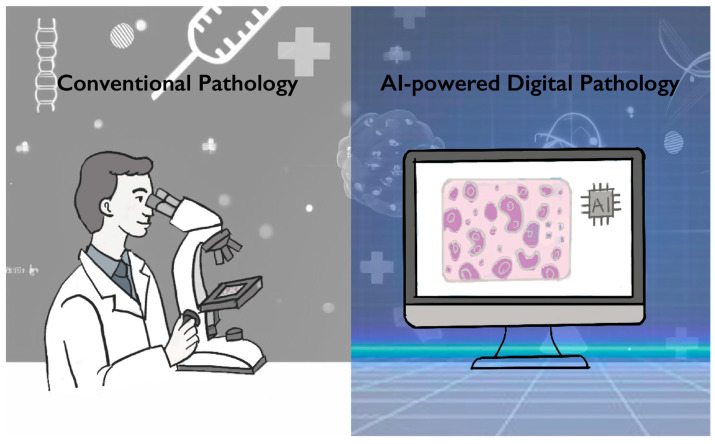
Conventional pathology versus AI-powered digital pathology. Conventional pathology relies on visual slide assessment by pathologists, which is time- and labor-intensive, whereas AI-powered digital pathology enables rapid and efficient whole-slide image analysis.

Recent advances in AI are reshaping how TILs are evaluated in pathology. Modern algorithms can accurately identify and quantify tumor nests, stromal areas, tumor cells, and lymphocytes across WSIs (Figure 1). Compared with traditional pathologist-based scoring, which is inherently subjective and time-consuming, automated approaches offer markedly improved consistency and reproducibility [41,42]. In melanoma, a multicenter study showed that AI-assisted TIL scoring correlated strongly with patient outcomes, reduced inter-observer variability, and enhanced prognostic reliability, underscoring its potential for clinical adoption [43].

### 3.2. Models and Approaches for TIL Detection

A range of machine learning architectures has been applied to WSIs to capture both morphological and spatial features of the TME:

- Convolutional Neural Networks (CNNs): CNNs remain the most widely used architecture in medical image analysis due to their ability to automatically learn hierarchical features from raw image data [44,45]. Through sequential convolutional, pooling, and fully connected layers, they convert pixel-level inputs into increasingly abstract representations [46]. Saltz et al. developed a CNN-based pipeline to quantify TIL density in The Cancer Genome Atlas (TCGA) cohort, demonstrating strong correlations with clinical outcomes across multiple tumor types [47]. CNNs are particularly effective for detecting cells and segmenting tissue regions, as they efficiently capture local textures, shapes, and structural patterns.
- Vision Transformers (ViTs): ViTs adapt the Transformer architecture, originally designed for natural language processing, to image analysis. By dividing images into patches and applying self-attention mechanisms, ViTs capture long-range spatial relationships while preserving positional information. In a large study of over 50,000 melanocytic lesions, a multi-ViT ensemble achieved high AUROC values and strong external generalizability, highlighting its clinical promise [48]. Unlike CNNs, which focus on local feature extraction, ViTs excel at modeling global tissue architecture and stromal-tumor organization [49].
- Graph Neural Networks (GNNs): GNNs represent histologic data as cell-level graphs, where nodes correspond to cells and edges encoding spatial or phenotypic relationships. Through iterative message passing, GNNs capture higher-order interactions within the TME that pixel-based methods may overlook. In melanoma, modeling cell-to-cell interactions with GNNs improved classification accuracy by about 10% compared with conventional machine-learning methods [50]. GNNs are particularly powerful for characterizing immune-cell clustering and tertiary lymphoid structures (TLS), offering insight into immune organization beyond simple density measures [51].

Each architecture has distinct strengths depending on the analytic goal. CNNs perform best for cellular-level tasks such as TIL detection or tumor segmentation, where fine morphological detail is most critical [46,47]. ViTs are advantageous when global tissue context or architectural organization drives biological behavior [48,49]. GNNs are most effective for studying spatial cell–cell interactions and immune ecology, as they operate directly on relational data rather than image grids [50,51]. Recently, hybrid frameworks combining CNN-based feature extraction with transformer- or graph-based reasoning have shown promise in integrating local and global information [52,53]. Future research using harmonized datasets and shared benchmarks will be essential for objectively comparing architectures and determining which best captures the multiscale complexity of melanoma histopathology.

### 3.3. Linking TIL Metrics to Therapy Response

As summarized in Table 1, studies across multiple cohorts and analytic approaches consistently show that AI-assisted TIL scoring is reproducible, aligns closely with pathologist grading, and provides additional prognostic value. Several algorithms have demonstrated that higher TIL density or eTIL% is significantly associated with improved disease-specific and overall survival, whereas low TIL density correlates with poorer outcomes. Emerging evidence also indicates that the spatial organization of lymphocytes, particularly their clustering near tumor cells, may carry prognostic relevance comparable to overall density. Collectively, these findings highlight AI-based TIL assessment as a promising biomarker for patient stratification in immunotherapy, particularly in neoadjuvant and adjuvant settings.

## 4. Spatial Distribution of TILs as an Advanced Biomarker

### 4.1. Concept of Inflamed, Excluded, and Desert Tumors

**Figure 2:**
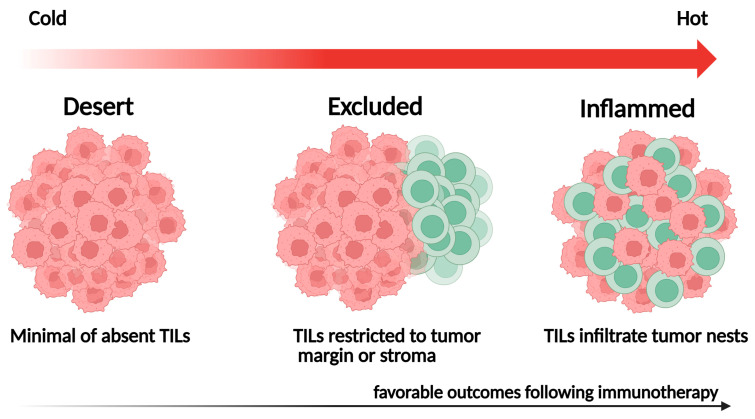
Representative spatial immune phenotypes of tumor-infiltrating lymphocytes (TILs).

The spatial distribution of TILs within the TME reflects distinct immunological phenotypes with important prognostic and predictive implications. Localization patterns, such as TIL density within tumor nests, stromal areas, or invasive margins, can influence antitumor immune responses and are closely associated with patient outcomes [61] (Figure 2):

- Inflamed tumors: Characterized by dense TIL infiltration within the tumor parenchyma, often indicating a pre-existing antitumor immune response and favorable outcomes following ICI therapy;
- Immune-excluded tumors: Contain abundant TILs confined to the surrounding stroma, reflecting stromal or vascular barriers that hinder immune-cell infiltration;
- Immune-desert tumors: Display minimal TIL presence in both tumor and stroma, signifying immune ignorance or tolerance and typically associated with poor outcomes.

In most AI-based studies, these immune phenotypes are defined by comparing the relative proportion or spatial distribution of lymphocytes in tumor nests versus stromal regions. However, quantitative thresholds for intratumoral-to-stromal TIL ratios vary across datasets and analytical methods, and no standardized cutoff has yet been established for defining inflamed, excluded, or desert phenotypes.

### 4.2. AI-Assisted Spatial Mapping

The manual classification of immune phenotypes is time-consuming and subject to inter-observer variability [62]. AI-based spatial analysis overcomes these challenges by integrating precise cell detection, tissue segmentation, and spatial statistics to provide a comprehensive understanding of the data. Notably, AI enables accurate localization of TILs within tumor parenchyma versus stroma, addressing a key limitation of conventional manual assessment. This capability allows standardized immune phenotyping across cohorts, facilitating large-scale, multicenter studies [63]. Moreover, these pipelines can concurrently analyze multiple components of TME at spatially resolved locations, enabling detailed immune phenotyping, data-driven determination of cutoffs, and greater potential for clinical translation [31,38,39]. Despite these advances, most melanoma studies still rely on density-based metrics, that, while reproducible and straightforward, fail to capture proximity, gradients, or topological relationships governing immune-tumor interactions.

Emerging frameworks such as Topological Tumor Graphs and the SPoTLIghT pipeline illustrate how topology- and graph-based methods can move beyond simple density measures to characterize the architectural and mechanistic complexity of the TME [64]. Cross-modal analyses have further linked histologic spatial patterns with molecular features. For example, Lapuente-Santana et al. developed spatial graphs using a model trained on integrated melanoma H&E slides and RNA-seq data to identify immune-infiltration patterns not detectable by molecular data alone, finally proposing a model that predicted patient prognosis [52].

### 4.3. Predictive and Prognostic Value for Immunotherapy

AI-driven spatial profiling of TILs has shown promise as a predictive biomarker for immunotherapy responses in various cancers. In non-small-cell lung cancer (NSCLC), Park et al. used AI to classify WSIs into three immune phenotypes, demonstrating that the inflamed type correlated with improved treatment response and survival, while Corredor et al. showed that graph-based SpaTIL features outperformed density measures in predicting recurrence [31,38]. Similarly, Lim et al. applied AI-powered spatial TIL analysis to colorectal cancer and confirmed its prognostic value [39]. In triple-negative breast cancer, Li et al. employed automated immunophenotyping to integrate spatial features that predicted a benefit from PD-L1-targeted therapy [62]. Across multiple tumor types, Shen et al. further demonstrated that an AI-defined inflamed phenotype consistently predicted favorable outcomes under immune checkpoint blockade [63].

In melanoma, most studies to date have focused primarily on TIL density rather than spatial context. Recently, Aung et al. demonstrated that spatially localized interferon-γ and chemokine-signaling programs coincided with CD8^+^-enriched tumor regions and more accurately predicted response to anti-PD-1 therapy than bulk transcriptomic profiles [65]. In parallel, preliminary evidence from Lim et al. suggested that AI-based immune phenotype profiling could stratify outcomes across melanoma subtypes, with the inflamed phenotype correlating with improved survival in cutaneous melanoma but limited predictive value in acral and mucosal melanoma [66]. Taken together, spatial frameworks, ranging from immune phenotype mapping to graph-based models, offer complementary insights into tumor-immune architecture. Establishing their predictive utility in melanoma remains a key objective for future research.

## 5. TLS: Ectopic Immune Niches

### 5.1. Definition, Cellular Composition, and Formation in Melanoma

**Figure 3:**
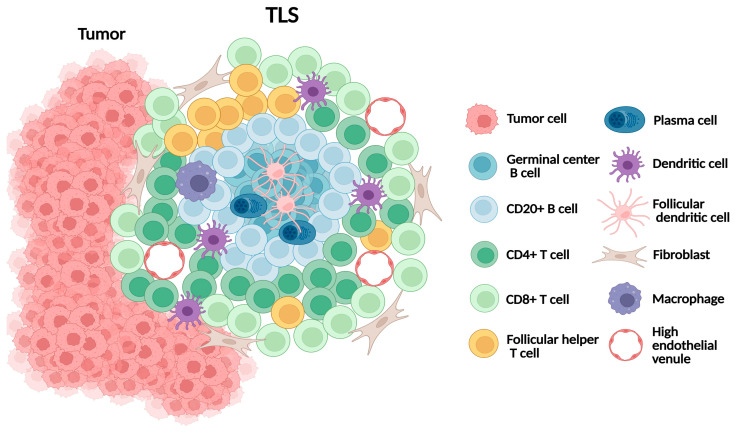
Schematic representation of tertiary lymphoid structure (TLS).

TLS are organized aggregates of immune cells that form ectopically in non-lymphoid tissues under conditions of chronic inflammation, including cancer [67] (Figure 3). They mirror many structural features of secondary lymphoid organs (SLOs), including B-cell follicles with germinal centers, T-cell-rich zones, follicular dendritic cells, and high endothelial venules (HEVs), which regulate lymphocyte trafficking. Unlike SLOs, TLSs lack a capsule and depend on stromal networks for their formation and maintenance. Mature TLSs exhibit a compartmentalized architecture, with CD20+ B-cell follicles at the core surrounded by CD3^+^ T-cell zones [68]. Several research groups have proposed multi-level TLS grading frameworks, including a four-tier framework defining absence, minimal, moderate, and extensive TLS presence [69,70]. However, these frameworks have not yet been universally validated or adopted.

In melanoma, TLSs are typically observed at the invasive margin or within the peritumoral stroma. Their formation is orchestrated by CAFs acting as lymphoid tissue organizer-like functions via TNF receptor signaling. CD8^+^ T cells contribute to stromal organization, while CXCL13-mediated B-cell recruitment and lymphotoxin-α_1_β_2_ sustain TLS expansion [71,72]. Collectively, TLSs provide a local environment that supports antigen presentation and the adaptive immune activation, making them critical immune niches within melanoma.

### 5.2. TLS and Survival/Immunotherapy Response in Melanoma

Accumulating evidence identifies TLSs as robust prognostic and predictive biomarkers across cancer types. Histopathologic and transcriptomic analyses consistently show that the presence, density, and maturation of TLSs are associated with favorable clinical outcomes, including longer overall survival (OS) and relapse-free survival (RFS). Patients with mature TLSs, characterized by germinal centers and active B-cell proliferation, exhibit stronger antitumor immunity than those with immature or absent TLSs [67,68,73]. Building on these findings, recent deep-learning frameworks have been developed to automatically detect and quantify TLSs from histologic and spatial data. Such models have been successfully applied across various tumor types, often leveraging large public datasets such as The Cancer Genome Atlas (TCGA) for pretraining and validation [74,75].

In melanoma, Cabrita et al. revealed that TLS-enriched tumors contained functional cytotoxic T cells and were associated with significantly improved survival, whereas TLS-negative tumors were dominated by dysfunctional T cells [76]. More recently, Wang et al. applied an AI-based segmentation pipeline, HookNet-TLS, to digitized H&E whole-slide images from the E1609 adjuvant melanoma trial (ipilimumab vs. interferon-α). This model automatically detected and quantified TLSs and germinal centers, enabling reproducible TLS mapping at scale and improving survival prediction in high-risk stage III/IV patients [77]. These algorithms were trained on manually annotated WSIs co-registered with immunohistochemical references (CD3, CD20, PNAd), providing ground-truth TLS labels for supervised learning.

Collectively, these findings reinforce TLSs as dynamic immunological hubs that shape antitumor immunity and serve as clinical biomarkers. AI-driven pathology now offers practical methods to integrate TLS detection and quantification into future immunotherapy trials, enabling reproducible and scalable assessment across large melanoma cohorts.

## 6. Current Challenges and Future Directions

### 6.1. Remaining Gaps in Current Evidence

Although most recent studies have analyzed digitized H&E WSIs, several key evidence gaps remain. First, the majority of cohorts are retrospective and derived from single or few institutions, with limited external validation. Analytic pipelines also vary markedly, including variations in tumor masking, stromal definitions, cell detection algorithms, and post-processing steps. These discrepancies are compounded by non-standardized TIL density thresholds, hindering reproducibility, cross-study comparability, and large-scale generalization [43,57,58,59,60]. Data quality and annotation reliability remain additional sources of bias. Variability in staining, scanner calibration, and artifact handling can influence model performance, while subjective or inconsistent labeling introduces systematic noise into training datasets. Annotation protocols often differ in how immune cells are defined, segmented, or selected for review, leading to biased ground truth and unstable model outputs. Standardized labeling guidelines, multicenter consensus datasets, and transparent annotation reporting will be essential to strengthen reproducibility.

Second, cohort diversity is also limited, with few Asian patients and an under-representation of acral melanoma. Arising on non-sun-exposed sites such as the palms, soles, and nail beds and exhibiting minimal ultraviolet mutational signatures, acral melanoma is a biologically distinct entity rather than a simple anatomic variant. Recurrent driver alterations include CDKN2A, KIT, MDM2, CCND1, CDK4, and PAK1, whereas BRAF and NRAS mutations are less frequent [78]. Its TME is generally less immune-infiltrated than that of other cutaneous melanoma, contributing to reduced responsiveness to immune checkpoint blockade. Nevertheless, higher TIL densities remain associated with improved survival [79]. Future research should incorporate more diverse populations and develop subtype-specific AI biomarkers that reflect these genomic and immunologic differences.

Finally, despite the availability of open-source pipelines such as ADTA and NN192, direct cross-evaluation on harmonized, multi-institutional datasets remains lacking [55,59,60]. A coordinated, community-wide effort is needed to establish shared reference datasets, standardized preprocessing protocols, and transparent benchmarking frameworks that enable consistent evaluation of computational pathology models. Such collaboration will promote methodological consistency, facilitate regulatory assessment, and accelerate clinical translation.

### 6.2. Technical Barriers and Multimodal Integration

While data quality and annotation bias remain major limitations, the next challenge lies in developing scalable and technically robust AI systems that can generalize across institutions. Such development depends on access to large, well-annotated datasets; however, manual labeling of immune cells in WSIs is remains labor-intensive and time-consuming [36,37]. Domain shift, where models trained on one institution’s data underperform externally, further limits scalability, driven by variations in staining, imaging platforms, or patient demographics. Addressing these issues requires harmonized slide preparation, as well as technical strategies such as transfer learning, federated learning, or domain adaptation [80,81].

Beyond these technical solutions, multimodal integration represents an important direction for future research. Ge et al. introduced a hybrid deep-learning framework that combines histology with spatial omics to delineate microanatomic and molecular interactions within the TME, while Gschwind and Ossowski integrated genomic and transcriptomic biomarkers to enhance prediction of anti-PD-1 response [53,82]. A complementary melanoma tumor-immune atlas further revealed coordinated spatial gene-expression gradients that define distinct immune niches within the TME [83]. Together, these approaches highlight how cross-modal fusion can yield composite biomarkers capturing both immune architecture and gene-expression activity, moving the filed closer to clinically deployable decision tools.

### 6.3. Translational and Practical Considerations

Looking ahead, research must transition from exploratory model development toward clinically oriented validation. Large, prospective, multi-institutional trials are essential for confirming the robustness of AI-derived immune metrics and establishing their role in patient selection for immunotherapy. Equally important is the establishment of standardized guidelines for pipeline design, cutoffs determination, and reporting practices to ensure reproducibility and comparability across studies. Expanding cohort diversity by including and subanalyzing under-represented melanoma subtypes such as acral melanoma will be critical for generalizability.

In addition to technical and clinical challenges, regulatory and operational barriers should also be addressed to support the integration of AI-powered digital pathology into existing clinical and laboratory workflows. These include the lack of standardized approval pathways for AI-assisted digital pathology systems, the need for external validation under regulatory oversight, and the requirement for traceable audit trails throughout development and deployment. Practical issues such as data governance, interoperability among platforms, and liability in diagnostic decision support must likewise be resolved before routine implementation.

Ultimately, clinical integration should focus not only on improving prognostic accuracy but also on demonstrating practical utility in therapy stratification, ideally through integration into biomarker-driven clinical trial designs. By aligning technical innovation with clinical relevance, AI-based pathology can progress from exploration to a reliable tool that enhance precision oncology in melanoma.

## 7. Conclusions

AI is transforming histopathology by enabling the automated quantification of TILs, spatially resolved immune profiling, and the digital detection of TLSs, offering new biomarkers that surpass the limits of conventional pathology. These approaches have demonstrated prognostic and predictive value, particularly for immunotherapy response. While AI-based tolls are not yet ready for routine clinical practice, continued refinement, combined with prospective validation and workflow integration, could make immune-informed digital pathology an integral component of precision oncology.

## Figures and Tables

**Table 1:** Summary of recent studies applying AI-based methods for TIL quantification in melanoma.

Study	Dataset (N)	AI Method	Target Features	Key Findings
Yang et al., 2021 [54] †	Text-based pathology reports (N = 2624)	Natural language processing (NLP) model (TIL extraction from medical records)	TIL grade (absent, nonbrisk, brisk)	Brisk TILs significantly associated with improved OS (HR = 0.63, *p* = 0.03, 5-year OS +14.2%); nonbrisk TILs not significant.
Moore et al., 2021 [55] †	H&E WSIs of primary melanoma (N = 145; multiple slides per tumor)	CNN (automated digital TIL analysis, ADTA; QuIP TIL CNN)	ADTA score = TIL-positive patches/total tumor patches	ADTA score correlated with pathologist grading (*p* < 0.001) and significantly predicted DSS (HR = 4.18, *p* = 0.006).
Chou et al., 2021 [56] †	H&E WSIs of primary melanoma (N = 453)	Machine learning algorithm for TIL quantification	TIL density	High TIL density predicted longer RFS (HR = 0.92 per 10% increase, *p* < 0.001) and OS (HR = 0.90, *p* = 0.002); manual grading did not predict RFS (*p* > 0.05).
Aung et al., 2022 [57] †	H&E WSIs of primary melanoma (N = 785)	Automated TIL quantification algorithm (eTILs, etTILs)	TIL density (eTIL%, etTILs)	eTIL% and etTILs significantly predicted OS (AUC = 0.77 and 0.793); identified high-risk stage II patients; TILs mainly CD3+/CD8+ or CD4+.
Ugolini et al., 2023 [58]	H&E WSIs of stage II–III melanoma with H&E and CD3 IHC (N = 307)	CNN (Inception-ResNet-v2)	TIL density (AI-TIL score = [TILs/(TILs + noTILs)] × 100)	High AI-TIL score predicted longer DFS (HR = 0.60, *p* = 0.013) and OS (HR = 0.59, *p* = 0.017).
Chatziioannou et al., 2023 [59] †	H&E WSIs of stage IB–IV melanoma (N = 641)	Neural network (NN192)	TIL density (eTIL score = [TILs/(TILs + tumor cells)] × 100%)	Low eTILs (≤16.6%) predicted poor prognosis; eTILs were lower in metastases (*p* = 0.0012). High eTILs (>12.2%) in therapy-naïve metastases associated with better survival under anti–PD-1 therapy (*p* = 0.037).
Tan et al., 2024 [60]	H&E WSIs of thin melanoma (≤1 mm) from 20-year cohort (N = 170; 85 fatal vs. 85 non-fatal)	Neural network (NN192)	eTIL% (electronic TIL density = TILs/[TILs + tumor cells])	Lowest eTIL% quartile associated with higher disease-specific mortality (OR = 4.77, *p* = 0.003); manual pathologist grading not predictive.
Aung et al., 2025 [43] †	H&E WSIs of primary melanoma (N = 208)	Machine learning algorithm (AI-assisted)	TIL density/stromal TILs	AI TIL scoring showed superior reproducibility vs. manual assessment (ICC > 0.90 vs. Kendall W 0.44) and predicted outcomes (median cutoff HR = 0.45, *p* = 0.005; 16.6% cutoff HR = 0.56, *p* = 0.04).

Abbreviations: AI, artificial Intelligence; Anti-PD-1, anti–programmed cell death protein 1 antibody; AUC, area under the curve; CNN, convolutional neural network; DFS, disease-free survival; DNN, deep neural network; DSS, disease-specific survival; eTIL, electronic TIL; etTIL, extended TIL; H&E, hematoxilyn and eosin; HR, hazard ratio; ICC, intraclass correlation coefficient; IHC, immunohistochemistry; OR, odds ratio; OS, overall survival; RFS, relapse-free survival; TIL, tumor-infiltrating lymphocyte; WSI, whole-slide image. Reported endpoints (OS, DSS, RFS, DFS, ORR) and time scales are presented as described in the original studies and are not directly comparable across cohorts. † Indicates studies that performed multivariable analyses adjusting for clinicopathologic variables such as Breslow thickness and ulceration. Studies without † either did not report or did not perform formal multivariable adjustment.

## Data Availability

The original contributions presented in this study are included in this article; further inquiries can be directed to the corresponding authors.

## References

[1] Glazer A.M., Winkelmann R.R., Farberg A.S., Rigel D.S. (2017). Analysis of Trends in US Melanoma Incidence and Mortality. JAMA Dermatol..

[2] Saginala K., Barsouk A., Aluru J.S., Rawla P., Barsouk A. (2021). Epidemiology of Melanoma. Med. Sci..

[3] Hamp A., Anderson J., Sivesind T.E., Szeto M.D., Hadjinicolaou A. (2021). From the Cochrane Library: Systemic Treatments for Metastatic Cutaneous Melanoma. JMIR Dermatol..

[4] Hoeijmakers L.L., Reijers I.L.M., Blank C.U. (2023). Biomarker-Driven Personalization of Neoadjuvant Immunotherapy in Melanoma. Cancer Discov..

[5] Shin S., Moon J., Oum C., Kim S., Cho S.I., Lim Y., Ock C.Y., Shin S. (2024). Discontinuation risk from adverse events: Immunotherapy alone vs. combined with chemotherapy: A systematic review and network meta-analysis. BMC Cancer.

[6] Alifu M., Tao M., Chen X., Chen J., Tang K., Tang Y. (2023). Checkpoint inhibitors as dual immunotherapy in advanced non-small cell lung cancer: A meta-analysis. Front. Oncol..

[7] Scobie M.R., Zhou K.I., Ahmed S., Kelley M.J. (2023). Utility of Tumor Mutational Burden as a Biomarker for Response to Immune Checkpoint Inhibition in the VA Population. JCO Precis. Oncol..

[8] Hossain S.M., Carpenter C., Eccles M.R. (2024). Genomic and Epigenomic Biomarkers of Immune Checkpoint Immunotherapy Response in Melanoma: Current and Future Perspectives. Int. J. Mol. Sci..

[9] Hamid O., Molinero L., Bolen C.R., Sosman J.A., Munoz-Couselo E., Kluger H.M., McDermott D.F., Powderly J.D., Sarkar I., Ballinger M. (2019). Safety, Clinical Activity, and Biological Correlates of Response in Patients with Metastatic Melanoma: Results from a Phase I Trial of Atezolizumab. Clin. Cancer Res..

[10] Johnson D.B., Frampton G.M., Rioth M.J., Yusko E., Xu Y., Guo X., Ennis R.C., Fabrizio D., Chalmers Z.R., Greenbowe J. (2016). Targeted Next Generation Sequencing Identifies Markers of Response to PD-1 Blockade. Cancer Immunol. Res..

[11] Roszik J., Haydu L.E., Hess K.R., Oba J., Joon A.Y., Siroy A.E., Karpinets T.V., Stingo F.C., Baladandayuthapani V., Tetzlaff M.T. (2016). Novel algorithmic approach predicts tumor mutation load and correlates with immunotherapy clinical outcomes using a defined gene mutation set. BMC Med..

[12] Samstein R.M., Lee C.H., Shoushtari A.N., Hellmann M.D., Shen R., Janjigian Y.Y., Barron D.A., Zehir A., Jordan E.J., Omuro A. (2019). Tumor mutational load predicts survival after immunotherapy across multiple cancer types. Nat. Genet..

[13] Ning B., Liu Y., Wang M., Li Y., Xu T., Wei Y. (2022). The Predictive Value of Tumor Mutation Burden on Clinical Efficacy of Immune Checkpoint Inhibitors in Melanoma: A Systematic Review and Meta-Analysis. Front. Pharmacol..

[14] Sinha N., Sinha S., Cheng K., Madan S., Schaffer A., Aldape K., Erez A., Ryan B.M., Ruppin E. (2021). Abstract 29: The recently approved high-TMB criteria may introduce a sex bias in response to PD1 inhibitors. Cancer Res..

[15] Madore J., Vilain R.E., Menzies A.M., Kakavand H., Wilmott J.S., Hyman J., Yearley J.H., Kefford R.F., Thompson J.F., Long G.V. (2015). PD-L1 expression in melanoma shows marked heterogeneity within and between patients: Implications for anti-PD-1/PD-L1 clinical trials. Pigment Cell Melanoma Res..

[16] Nebhan C.A., Johnson D.B. (2020). Predictive biomarkers of response to immune checkpoint inhibitors in melanoma. Expert Rev. Anticancer Ther..

[17] Song K.Y., Desar S., Pengo T., Shanley R., Giubellino A. (2020). Correlation of MET and PD-L1 Expression in Malignant Melanoma. Cancers.

[18] Ma W., Liu W., Zhong J., Zou Z., Lin X., Sun W., Hu T., Xu Y., Chen Y. (2024). Advances in predictive biomarkers for melanoma immunotherapy. Holist. Integr. Oncol..

[19] Morrison C., Pabla S., Conroy J.M., Nesline M.K., Glenn S.T., Dressman D., Papanicolau-Sengos A., Burgher B., Andreas J., Giamo V. (2018). Predicting response to checkpoint inhibitors in melanoma beyond PD-L1 and mutational burden. J. Immunother. Cancer.

[20] Ciresan D.C., Giusti A., Gambardella L.M., Schmidhuber J. (2013). Mitosis detection in breast cancer histology images with deep neural networks. Medical Image Computing and Computer-Assisted Intervention—MICCAI 2013.

[21] Esteva A., Kuprel B., Novoa R.A., Ko J., Swetter S.M., Blau H.M., Thrun S. (2017). Dermatologist-level classification of skin cancer with deep neural networks. Nature.

[22] Noorbakhsh-Sabet N., Zand R., Zhang Y., Abedi V. (2019). Artificial Intelligence Transforms the Future of Health Care. Am. J. Med..

[23] Phillips S.P., Spithoff S., Simpson A. (2022). Artificial intelligence and predictive algorithms in medicine: Promise and problems. Can. Fam. Physician.

[24] Rodriguez-Ruiz A., Lang K., Gubern-Merida A., Broeders M., Gennaro G., Clauser P., Helbich T.H., Chevalier M., Tan T., Mertelmeier T. (2019). Stand-Alone Artificial Intelligence for Breast Cancer Detection in Mammography: Comparison with 101 Radiologists. J. Natl. Cancer Inst..

[25] Niazi M.K.K., Parwani A.V., Gurcan M.N. (2019). Digital pathology and artificial intelligence. Lancet Oncol..

[26] Pitt J.M., Marabelle A., Eggermont A., Soria J.C., Kroemer G., Zitvogel L. (2016). Targeting the tumor microenvironment: Removing obstruction to anticancer immune responses and immunotherapy. Ann. Oncol..

[27] Li Y.R., Fang Y., Lyu Z., Zhu Y., Yang L. (2023). Exploring the dynamic interplay between cancer stem cells and the tumor microenvironment: Implications for novel therapeutic strategies. J. Transl. Med..

[28] Lv B., Wang Y., Ma D., Cheng W., Liu J., Yong T., Chen H., Wang C. (2022). Immunotherapy: Reshape the Tumor Immune Microenvironment. Front. Immunol..

[29] Rohaan M.W., Borch T.H., van den Berg J.H., Met O., Kessels R., Geukes Foppen M.H., Stoltenborg Granhoj J., Nuijen B., Nijenhuis C., Jedema I. (2022). Tumor-Infiltrating Lymphocyte Therapy or Ipilimumab in Advanced Melanoma. N. Engl. J. Med..

[30] Maibach F., Sadozai H., Seyed Jafari S.M., Hunger R.E., Schenk M. (2020). Tumor-Infiltrating Lymphocytes and Their Prognostic Value in Cutaneous Melanoma. Front. Immunol..

[31] Park S., Ock C.Y., Kim H., Pereira S., Park S., Ma M., Choi S., Kim S., Shin S., Aum B.J. (2022). Artificial Intelligence-Powered Spatial Analysis of Tumor-Infiltrating Lymphocytes as Complementary Biomarker for Immune Checkpoint Inhibition in Non-Small-Cell Lung Cancer. J. Clin. Oncol..

[32] Sun R., Limkin E.J., Vakalopoulou M., Dercle L., Champiat S., Han S.R., Verlingue L., Brandao D., Lancia A., Ammari S. (2018). A radiomics approach to assess tumour-infiltrating CD8 cells and response to anti-PD-1 or anti-PD-L1 immunotherapy: An imaging biomarker, retrospective multicohort study. Lancet Oncol..

[33] Amaria R.N., Reddy S.M., Tawbi H.A., Davies M.A., Ross M.I., Glitza I.C., Cormier J.N., Lewis C., Hwu W.J., Hanna E. (2018). Neoadjuvant immune checkpoint blockade in high-risk resectable melanoma. Nat. Med..

[34] Fu Q., Chen N., Ge C., Li R., Li Z., Zeng B., Li C., Wang Y., Xue Y., Song X. (2019). Prognostic value of tumor-infiltrating lymphocytes in melanoma: A systematic review and meta-analysis. Oncoimmunology.

[35] Ladanyi A. (2015). Prognostic and predictive significance of immune cells infiltrating cutaneous melanoma. Pigment Cell Melanoma Res..

[36] Aeffner F., Zarella M.D., Buchbinder N., Bui M.M., Goodman M.R., Hartman D.J., Lujan G.M., Molani M.A., Parwani A.V., Lillard K. (2019). Introduction to Digital Image Analysis in Whole-slide Imaging: A White Paper from the Digital Pathology Association. J. Pathol. Inform..

[37] Chen C.L., Chen C.C., Yu W.H., Chen S.H., Chang Y.C., Hsu T.I., Hsiao M., Yeh C.Y., Chen C.Y. (2021). An annotation-free whole-slide training approach to pathological classification of lung cancer types using deep learning. Nat. Commun..

[38] Corredor G., Wang X., Zhou Y., Lu C., Fu P., Syrigos K., Rimm D.L., Yang M., Romero E., Schalper K.A. (2019). Spatial Architecture and Arrangement of Tumor-Infiltrating Lymphocytes for Predicting Likelihood of Recurrence in Early-Stage Non-Small Cell Lung Cancer. Clin. Cancer Res..

[39] Lim Y., Choi S., Oh H.J., Kim C., Song S., Kim S., Song H., Park S., Kim J.W., Kim J.W. (2023). Artificial intelligence-powered spatial analysis of tumor-infiltrating lymphocytes for prediction of prognosis in resected colon cancer. NPJ Precis. Oncol..

[40] Roxburgh C.S., McMillan D.C. (2012). The role of the in situ local inflammatory response in predicting recurrence and survival in patients with primary operable colorectal cancer. Cancer Treat. Rev..

[41] Albusayli R., Graham J.D., Pathmanathan N., Shaban M., Raza S.E.A., Minhas F., Armes J.E., Rajpoot N. (2023). Artificial intelligence-based digital scores of stromal tumour-infiltrating lymphocytes and tumour-associated stroma predict disease-specific survival in triple-negative breast cancer. J. Pathol..

[42] Rakaee M., Adib E., Ricciuti B., Sholl L.M., Shi W., Alessi J.V., Cortellini A., Fulgenzi C.A.M., Viola P., Pinato D.J. (2023). Association of Machine Learning-Based Assessment of Tumor-Infiltrating Lymphocytes on Standard Histologic Images with Outcomes of Immunotherapy in Patients with NSCLC. JAMA Oncol..

[43] Aung T.N., Liu M., Su D., Shafi S., Boyaci C., Steen S., Tsiknakis N., Vidal J.M., Maher N., Micevic G. (2025). Pathologist-Read vs AI-Driven Assessment of Tumor-Infiltrating Lymphocytes in Melanoma. JAMA Netw. Open.

[44] Kong X.Y., Zhao X.S., Sun X.H., Wang P., Wu Y., Peng R.Y., Zhang Q.Y., Wang Y.Z., Li R., Yang Y.H. (2023). Classification of Glomerular Pathology Images in Children Using Convolutional Neural Networks with Improved SE-ResNet Module. Interdiscip. Sci. Comput. Life Sci..

[45] Li Y.X., Chen F., Shi J.J., Huang Y.L., Wang M. (2023). Convolutional Neural Networks for Classifying Cervical Cancer Types Using Histological Images. J. Digit. Imaging.

[46] Khosravi P., Kazemi E., Imielinski M., Elemento O., Hajirasouliha I. (2018). Deep Convolutional Neural Networks Enable Discrimination of Heterogeneous Digital Pathology Images. eBioMedicine.

[47] Saltz J., Gupta R., Hou L., Kurc T., Singh P., Nguyen V., Samaras D., Shroyer K.R., Zhao T., Batiste R. (2018). Spatial Organization and Molecular Correlation of Tumor-Infiltrating Lymphocytes Using Deep Learning on Pathology Images. Cell Rep..

[48] Lucassen R.T., Stathonikos N., Breimer G.E., Veta M., Blokx W.A. (2025). Artificial intelligence-based triaging of cutaneous melanocytic lesions. npj Biomed. Innov..

[49] Katayama A., Aoki Y., Watanabe Y., Horiguchi J., Rakha E.A., Oyama T. (2024). Current status and prospects of artificial intelligence in breast cancer pathology: Convolutional neural networks to prospective Vision Transformers. Int. J. Clin. Oncol..

[50] Monroy L.C.R., Rist L., Eberhardt M., Ostalecki C., Baur A., Vera J., Breininger K., Maier A. Employing graph representations for cell-level characterization of melanoma MELC samples. Proceedings of the 2023 IEEE 20th International Symposium on Biomedical Imaging (ISBI).

[51] Gogoshin G., Rodin A.S. (2023). Graph Neural Networks in Cancer and Oncology Research: Emerging and Future Trends. Cancers.

[52] Lapuente-Santana O., Kant J., Eduati F. (2024). Integrating histopathology and transcriptomics for spatial tumor microenvironment profiling in a melanoma case study. NPJ Precis. Oncol..

[53] Ge Y., Leng J., Tang Z., Wang K., U K., Zhang S.M., Han S., Zhang Y., Xiang J., Yang S. (2025). Deep Learning-Enabled Integration of Histology and Transcriptomics for Tissue Spatial Profile Analysis. Research.

[54] Yang J., Lian J.W., Chin Y.H., Wang L., Lian A., Murphy G.F., Zhou L. (2021). Assessing the Prognostic Significance of Tumor-Infiltrating Lymphocytes in Patients with Melanoma Using Pathologic Features Identified by Natural Language Processing. JAMA Netw. Open.

[55] Moore M.R., Friesner I.D., Rizk E.M., Fullerton B.T., Mondal M., Trager M.H., Mendelson K., Chikeka I., Kurc T., Gupta R. (2021). Automated digital TIL analysis (ADTA) adds prognostic value to standard assessment of depth and ulceration in primary melanoma. Sci. Rep..

[56] Chou M., Illa-Bochaca I., Minxi B., Darvishian F., Johannet P., Moran U., Shapiro R.L., Berman R.S., Osman I., Jour G. (2021). Optimization of an automated tumor-infiltrating lymphocyte algorithm for improved prognostication in primary melanoma. Mod. Pathol..

[57] Aung T.N., Shafi S., Wilmott J.S., Nourmohammadi S., Vathiotis I., Gavrielatou N., Fernandez A., Yaghoobi V., Sinnberg T., Amaral T. (2022). Objective assessment of tumor infiltrating lymphocytes as a prognostic marker in melanoma using machine learning algorithms. eBioMedicine.

[58] Ugolini F., De Logu F., Iannone L.F., Brutti F., Simi S., Maio V., de Giorgi V., Maria di Giacomo A., Miracco C., Federico F. (2023). Tumor-Infiltrating Lymphocyte Recognition in Primary Melanoma by Deep Learning Convolutional Neural Network. Am. J. Pathol..

[59] Chatziioannou E., Rossner J., Aung T.N., Rimm D.L., Niessner H., Keim U., Serna-Higuita L.M., Bonzheim I., Kuhn Cuellar L., Westphal D. (2023). Deep learning-based scoring of tumour-infiltrating lymphocytes is prognostic in primary melanoma and predictive to PD-1 checkpoint inhibition in melanoma metastases. eBioMedicine.

[60] Tan S.X., Aung T.N., Claeson M., Acs B., Zhou C., Brown S., Lambie D., Baade P.D., Pandeya N., Soyer H.P. (2024). Automated scoring of tumor-infiltrating lymphocytes informs risk of death from thin melanoma: A nested case-case study. J. Am. Acad. Dermatol..

[61] Zheng S., Wang W., Shen L., Yao Y., Xia W., Ni C. (2024). Tumor battlefield within inflamed, excluded or desert immune phenotypes: The mechanisms and strategies. Exp. Hematol. Oncol..

[62] Li X., Eastham J., Giltnane J.M., Zou W., Zijlstra A., Tabatsky E., Banchereau R., Chang C.W., Nabet B.Y., Patil N.S. (2024). Automated tumor immunophenotyping predicts clinical benefit from anti-PD-L1 immunotherapy. J. Pathol..

[63] Shen J., Choi Y.L., Lee T., Kim H., Chae Y.K., Dulken B.W., Bogdan S., Huang M., Fisher G.A., Park S. (2024). Inflamed immune phenotype predicts favorable clinical outcomes of immune checkpoint inhibitor therapy across multiple cancer types. J. Immunother. Cancer.

[64] Failmezger H., Muralidhar S., Rullan A., de Andrea C.E., Sahai E., Yuan Y. (2020). Topological Tumor Graphs: A Graph-Based Spatial Model to Infer Stromal Recruitment for Immunosuppression in Melanoma Histology. Cancer Res..

[65] Aung T.N., Warrell J., Martinez-Morilla S., Gavrielatou N., Vathiotis I., Yaghoobi V., Kluger H.M., Gerstein M., Rimm D.L. (2024). Spatially Informed Gene Signatures for Response to Immunotherapy in Melanoma. Clin. Cancer Res..

[66] Hwang S., Ryu H.J., Kim Y., Cho S.I., Oum C., Puche A.V., Lee J., Kim K.H., Chung K.Y., Shin S.J. (2024). Immune phenotype profiling based on anatomic origin of melanoma and impact on clinical outcomes of immune checkpoint inhibitor treatment. J. Clin. Oncol..

[67] Sautes-Fridman C., Petitprez F., Calderaro J., Fridman W.H. (2019). Tertiary lymphoid structures in the era of cancer immunotherapy. Nat. Rev. Cancer.

[68] Zhang Q., Wu S. (2022). Tertiary lymphoid structures are critical for cancer prognosis and therapeutic response. Front. Immunol..

[69] Su G.L., Zhang M.J., Li H., Sun Z.J. (2025). Dissecting Tertiary Lymphoid Structures in Cancer: Maturation, Localization and Density. Theranostics.

[70] Rakaee M., Kilvaer T.K., Jamaly S., Berg T., Paulsen E.E., Berglund M., Richardsen E., Andersen S., Al-Saad S., Poehl M. (2021). Tertiary lymphoid structure score: A promising approach to refine the TNM staging in resected non-small cell lung cancer. Br. J. Cancer.

[71] Yoshimitsu M., Nakamura M., Kano S., Magara T., Kato H., Sakai A., Sugiyama M., Mizokami M., Morita A. (2025). CXCL13 and CCL21 Induce Tertiary Lymphoid Structures and Enhance the Efficacy of Immunotherapy for Melanoma. Cancer Sci..

[72] Rodriguez A.B., Peske J.D., Woods A.N., Leick K.M., Mauldin I.S., Meneveau M.O., Young S.J., Lindsay R.S., Melssen M.M., Cyranowski S. (2021). Immune mechanisms orchestrate tertiary lymphoid structures in tumors via cancer-associated fibroblasts. Cell Rep..

[73] Sautes-Fridman C., Lawand M., Giraldo N.A., Kaplon H., Germain C., Fridman W.H., Dieu-Nosjean M.C. (2016). Tertiary Lymphoid Structures in Cancers: Prognostic Value, Regulation, and Manipulation for Therapeutic Intervention. Front. Immunol..

[74] van Rijthoven M., Obahor S., Pagliarulo F., van den Broek M., Schraml P., Moch H., van der Laak J., Ciompi F., Silina K. (2024). Multi-resolution deep learning characterizes tertiary lymphoid structures and their prognostic relevance in solid tumors. Commun. Med..

[75] Li Z., Jiang Y., Li B., Han Z., Shen J., Xia Y., Li R. (2023). Development and Validation of a Machine Learning Model for Detection and Classification of Tertiary Lymphoid Structures in Gastrointestinal Cancers. JAMA Netw. Open.

[76] Cabrita R., Lauss M., Sanna A., Donia M., Skaarup Larsen M., Mitra S., Johansson I., Phung B., Harbst K., Vallon-Christersson J. (2020). Tertiary lymphoid structures improve immunotherapy and survival in melanoma. Nature.

[77] Tarhini A.A., Li T., Tao R., Lee S.J., Eljilany I., Hodi F.S., Streicher H., Karunamurthy A., Kirkwood J.M., Wang X. (2025). Abstract 3358: Tertiary lymphoid structures (TLS) estimated by AI tools from digital H&E slides significantly enhance survival prediction in high-risk AJCC stages III/IV melanoma. Cancer Res..

[78] Broit N., Johansson P.A., Rodgers C.B., Walpole S.T., Hayward N.K., Pritchard A.L. (2022). Systematic review and meta-analysis of genomic alterations in acral melanoma. Pigment Cell Melanoma Res..

[79] Borkowska A.M., Szumera-Cieckiewicz A., Chraszczewska M., Sokol K., Goryn T., Rutkowski P.L. (2021). Clinical Significance of Tumor Microenvironment in Acral Melanoma: A Large Single-Institution Study of Caucasians. J. Clin. Med..

[80] Asadi-Aghbolaghi M., Darbandsari A., Zhang A., Contreras-Sanz A., Boschman J., Ahmadvand P., Kobel M., Farnell D., Huntsman D.G., Churg A. (2024). Learning generalizable AI models for multi-center histopathology image classification. npj Precis. Oncol..

[81] Haggenmuller S., Schmitt M., Krieghoff-Henning E., Hekler A., Maron R.C., Wies C., Utikal J.S., Meier F., Hobelsberger S., Gellrich F.F. (2024). Federated Learning for Decentralized Artificial Intelligence in Melanoma Diagnostics. JAMA Dermatol..

[82] Gschwind A., Ossowski S. (2025). AI Model for Predicting Anti-PD1 Response in Melanoma Using Multi-Omics Biomarkers. Cancers.

[83] Chelebian E., Avenel C., Wahlby C. (2025). Combining spatial transcriptomics with tissue morphology. Nat. Commun..

